# Awareness of Prevention Strategies and Willingness to Use Preexposure Prophylaxis in Brazilian Men Who Have Sex With Men Using Apps for Sexual Encounters: Online Cross-Sectional Study

**DOI:** 10.2196/publichealth.8997

**Published:** 2018-01-22

**Authors:** Thiago Silva Torres, Raquel Brandini De Boni, Mauricio TL de Vasconcellos, Paula Mendes Luz, Brenda Hoagland, Ronaldo Ismerio Moreira, Valdilea Gonçalves Veloso, Beatriz Grinsztejn

**Affiliations:** ^1^ Instituto Nacional de Infectologia Evandro Chagas, Fundação Oswaldo Cruz Rio de Janeiro Brazil; ^2^ Escola Nacional de Ciências Estatísticas Rio de Janeiro Brazil

**Keywords:** HIV, prevention, MSM, app, internet, PrEP, Brazil, Latin America

## Abstract

**Background:**

Geosocial networking (GSN) smartphone apps are becoming the main venue for sexual encounters among Brazilian men who have sex with men (MSM). To address the increased HIV incidence in this population, preexposure prophylaxis (PrEP) was recently implemented in the Brazilian public health system in the context of combined HIV prevention.

**Objective:**

This study aimed to describe the characteristics of MSM using GSN apps for sexual encounters, their awareness of prevention strategies, and willingness to use PrEP.

**Methods:**

This study was an online cross-sectional study conducted in 10 Brazilian state capitals from July 1 to July 31, 2016. The questionnaire was programmed on SurveyGizmo and advertised in two GSN apps used by MSM to find sexual partners (Hornet and Grindr). Inclusion criteria were >18 years of age, cisgender men, with an HIV-negative status. Eligible individuals answered questions on: demographics; behavior; and knowledge, preferences, and willingness to use PrEP, nonoccupational postexposure prophylaxis (nPEP), HIV self-testing (HIVST), and condoms. Logistic regression modeling was performed to assess the factors associated with daily oral PrEP willingness.

**Results:**

During the study period, 8885 individuals provided consent and started the questionnaire. Of these, 23.05% (2048/8885) were ineligible, 6837 (6837/8885, 76.94%) initiated, and 5065 (5065/8885, 57.00%) completed the entire questionnaire and were included in the present analysis. Median age was 30 years (interquartile range: 25-36), most self-declared as MSM (4991/5065, 98.54%), white (3194/5065, 63.06%), middle income (2148/5065, 42.41%), and had 12 or more years of schooling (3106/5062, 61.36%). The majority of MSM (3363/5064, 66.41%) scored >10 points (high risk) on The HIV Incidence Risk for MSM Scale, but only 21.39% (1083/5064) had a low perceived likelihood of getting HIV in the next year. Daily use of apps for sex was reported by 35.58% (1798/5054). Most MSM (4327/5065, 85.43%) reported testing for HIV at least once in their lifetime and 9.16% (464/5065) used nPEP in the previous year. PrEP, nPEP, and HIVST awareness was reported by 57.89% (2932/5065), 57.39% (2907/5065), and 26.57% (1346/5065) of participants, respectively. Half of all respondents (2653/5065, 52.38%) were willing to use daily oral PrEP, and this finding was associated with higher numbers of male sexual partners (adjusted odds ratio [AOR] 1.26, 95% CI 1.09-1.47), condomless receptive anal intercourse (AOR 1.27, 95% CI 1.12-1.44), sex with HIV-positive partner versus no HIV-positive partner (one HIV-positive partner: AOR 1.36, 95% CI 1.11-1.67), daily use of apps for sexual encounters (AOR 1.48, 95% CI 1.17-1.87), high and unknown perceived likelihood of getting HIV in the next year (AOR 1.72, 95% CI 1.47-2.02 and AOR 1.39, 95% CI 1.13-1.70), sexually transmitted infection diagnosis (AOR 1.25, 95% CI 1.03-1.51), stimulant use (AOR 1.24, 95% CI 1.07-1.43), PrEP awareness (AOR 1.48, 95% CI 1.30-1.70), and unwillingness to use condoms (AOR 1.16, 95% CI 1.00-1.33).

**Conclusions:**

Our results evidenced high-risk scores in the studied population, suggesting the importance of PrEP use. Those individuals presenting risky sexual behaviors were more willing to use PrEP. Nonetheless, only 58% (2932/5065) of individuals had heard about this prevention strategy. Efforts to increase awareness of new prevention strategies are needed, and mobile health tools are a promising strategy to reach MSM.

## Introduction

Brazil has the largest population of individuals living with HIV and acquired immune deficiency syndrome (AIDS) in Latin America [1], representing a concentrated epidemic with an estimated HIV prevalence of 0.6% in the general population (0.4% among women and 0.8% among men), and a 14.2% prevalence among men who have sex with men (MSM) [2]. Geographic differences have been reported in HIV prevalence among MSM (ranging from 5.2% [Recife] to 23.7% [Brasília]) in a respondent-driven sample survey conducted in 10 cities [2]. In 2016, approximately 60% of reported HIV infections were attributed to male-to-male sexual contact, although MSM represent only 3.5% of the Brazilian population [3,4]. New infections in this population continue to rise, especially among young people (24 years-old or less) [3].

Preexposure prophylaxis (PrEP) with tenofovir/emtricitabine is now a key component of prevention packages for MSM. The efficacy of treatment for this population has been shown with both once daily and on-demand use in clinical trials and open-label studies [5-9], and demonstration studies have been successfully implemented in different settings [10-13]. Results show that willingness to use PrEP among MSM may vary across different geographic locations, ranging from 32% to 92% [14-28].

With expanded access to the Internet via mobile phones, geosocial networking (GSN) smartphone apps are becoming the main venue for casual sexual encounters [29-32]. MSM report logging into GSN apps at least three times per day, with an average of 12 minutes spent per log-in [33,34]. These new technologies have generated quicker and easier ways for MSM to meet potential partners, and may facilitate the users’ ability to have multiple concurrent partners, thereby increasing their risk for acquiring HIV [32,35-37]. Men who use the Internet to meet other men may present a different behavioral profile than men who meet men in physical venues. For instance, MSM who do not identify as such would be excluded from venue-sampling [38]. Therefore, a better understanding of the profile of MSM who use apps for sexual encounters is needed in order to design tailored, combined prevention interventions. This study aimed to describe the characteristics of Brazilian MSM using two GSN apps for sexual encounters, including risk behavior for HIV infection and their awareness of prevention strategies. In addition, factors associated with daily oral PrEP willingness were assessed.

## Methods

### Study Design

This was a cross-sectional online study targeting MSM from 10 Brazilian capitals (two from each Brazilian geographical region): Belém and Manaus (North); Salvador and Recife (Northeast); Brasília and Goiânia (Central-West); Florianópolis and Porto Alegre (South); and Rio de Janeiro and São Paulo (Southeast). According to the 2010 Brazilian Population Census, these are the cities with the greatest number of MSM couples from each region [39]. Individuals who met eligibility criteria (age >18 years, cisgender men, and HIV-uninfected) and who acknowledged reading the informed consent were directed to the online questionnaire.

The questionnaire was programmed on SurveyGizmo [40] and the project was advertised on two GSN apps: Hornet [41] and Grindr [42]. Hornet users received an inbox message with a link to the survey on July 1, 2016 and July 22, 2016. Grindr users received a pop-up advertisement in July 2016 (days 9, 13, 17, 23, 27). In addition, a total of 5,050,000 banners were advertised on the Grindr interface. The questionnaire remained open from July 1, 2016 to July 31, 2016. No incentives were provided for answering the survey.

### Variables

#### Sociodemographics

Age was categorized into three brackets: (1) 18-to-24 years; (2) 25-to-34 years; and (3) >35 years. Skin color/race was dichotomized into "white" or "non-white" (Black, Asian, Native American, mixed-race, or don’t know). Schooling was dichotomized into "<12 years" and ">12 years" (12 years is equivalent to completing high school education in Brazil). Family monthly income was grouped into "<3" (low income), "4-to-10" (middle income), and ">10" minimum (high income) wages (Brazilian minimum wage was 880 BRL or US $267 dollars in July 2016). Sexual orientation was dichotomized into "MSM" (homosexual/gay/bisexual) or "other", and the options for a question regarding their friends with the same sexual orientation were "none", "a few", and "majority". Individuals were asked if they had a steady partner and the options were "yes/no", "male" and "female".

#### Substance Use

Binge drinking [43] was evaluated with the question, “In the last 6 months, did you drink 5 or more drinks in a couple of hours?” *Any substance use* in the prior six months considered the use of any of the following: tobacco (cigarettes), stimulants (cocaine, crack, amphetamines), 4-hydroxybutanoic acid (GHB), marijuana or hash, and hallucinogens (solvents, lysergic acid diethylamide, ketamine), which were displayed in a predefined list of all substances.

#### Use of Apps

The variable “Apps” was created based on the question, “Where did you hear about this questionnaire?” (with response format as open text) and was categorized into *Hornet*, *Grindr*, and *other*. Although the project was advertised only on Hornet and Grindr, the link to the survey could be copied and exchanged through other media (ie, email, Facebook, Whatsapp). The use of apps for sexual encounters was categorized into "never", "sometimes" (once a month, once a week, only on weekends), "daily", and "only when traveling or vacations".

#### Sexual Behavior, Sexually Transmitted Diseases, and Nonoccupational Postexposure Prophylaxis

Sexual behavior in the last six months was assessed through the following questions: number of partners (0-5, >6-10, and more than 10); condomless receptive anal sex (yes or no); sex with HIV-positive partner (no, one, more than one, or unknown); number of insertive anal intercourses with HIV-positive partner (no, 1-4 intercourses, 5 or more intercourses, or unknown). These questions belong to *The HIV Incidence Risk for MSM Scale*, which is a 7-item questionnaire developed by Smith et al [44] to predict HIV seroconversion among MSM. It is recommended by the Centers for Disease Control and Prevention (CDC) to screen individuals who should be evaluated to receive PrEP [45]. Scores >10 were considered “high risk” [44,45]. Sex for money and sexually transmitted infections (STIs; syphilis, gonorrhea or rectal chlamydia) were dichotomized into "yes" or "no". Participants were asked if they used nonoccupational postexposure prophylaxis (nPEP) in the past 12 months.

#### Perceived Likelihood of Getting HIV in Next Year and HIV Testing

Perceived likelihood of getting HIV in the next year was assessed through the question, “What is your chance of getting HIV in the next year?” with possible options grouped into "Low" (None/Low), "High" (Some/High/Certainly) and "unknown" [21]. Additionally, individuals were asked about previous HIV testing (never, once in lifetime, once a year, more than once a year, every time I am exposed, or sporadically) and preferences (reasons for not testing, best place for testing, best way to obtain HIV self-testing (HIVST) if available at the Brazilian public health system; SUS), as well as if they know someone living with HIV (Yes/No).

#### Awareness and Willingness to Use HIV Prevention Measures

Awareness of HIV prevention measures including daily PrEP, postexposure prophylaxis (PEP), and HIVST were assessed through the question, ‘‘Have you ever heard of...to prevent HIV infection?’’ Willingness to use HIV prevention measures including condoms, daily oral PrEP, and PEP was defined as the "High interest" option on a four-point Likert scale through the question, ‘‘In case it was available at SUS, what level of interest would you have in using...for preventing HIV?” A brief explanation on the preventive measures was provided before these questions were asked. These questions have previously been used by our research team to describe PrEP awareness and willingness [21]. Finally, we assessed individuals’ willingness to use PrEP and HIV self-testing even if they had to pay for it.

Willingness to use different PrEP regimens was assessed with the following question, “Which of the following PrEP regimens would you take if available?” Participants could select one or more of the following options: "PrEP on demand" (two pills 24 hours before intercourse and one pill 24 hours and 48 hours after), "injection PrEP" (injection drug every 2 months), or "would never use PrEP".

### Ethical Issues

Instituto Nacional de Infectologia Evandro Chagas INI-FIOCRUZ institutional review board approved this study (#51595815.7.0000.5262 at ‘‘Plataforma Brasil’’) in accordance with all applicable regulations, and all study participants digitally signed an informed consent form. No identification of participants was collected.

### Statistical Analysis

Characteristics, attitudes, and behaviors of the participants were described by their absolute and relative frequencies. Chi-square tests were used to compare characteristics of the individuals who completed and did not complete the questionnaire. A bivariate logistic regression analysis was performed to explore factors associated with willingness to use oral daily PrEP (odds ratios [OR]). Afterwards, a backwards stepwise logistic regression modeling approach was used to identify the factors independently associated with daily oral PrEP willingness [46]. Variables with *P*<.25 in bivariate analysis models were included in the initial multivariate model, and subsequently excluded if their P-value was >.05. The final multivariate model included both variables that remained significant (at a 5% significance threshold) and those found to be confounders (ie, those that changed the OR estimate of any of the remaining variables by more than 10%). Age, color/race, and schooling were defined a priori as confounders and were kept in the final multivariate model irrespectively of significance level (adjusted odds ratios [AOR]). Analyses were performed using PROC GENMOD available in the Software SAS [47].

## Results

**Figure 1:**
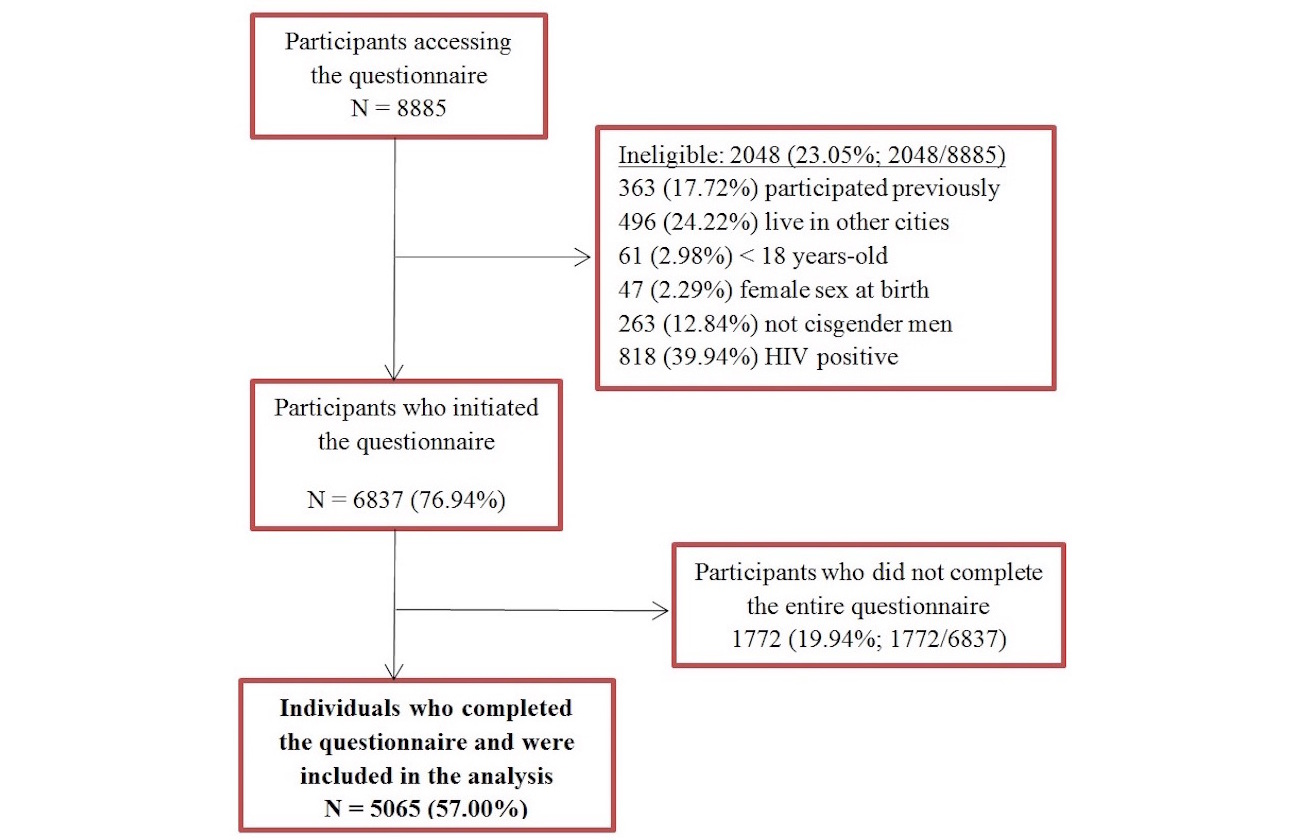
Study flow-chart.

During the 30 days of the online survey, 8885 individuals provided informed consent. Of these, 23.05% (2048/8885) were ineligible, 6837 (6837/8885, 76.94%) initiated the questionnaire, and 5065 (5065/8885, 57.00%) completed the questionnaire and were included in the present analysis (Figure 1). Differences among those who did not complete the questionnaire (n=1772) and those who completed the questionnaire (n=5065) are presented in Table 1.

Among all individuals who accessed the questionnaire and completed questions regarding HIV serostatus (n=6664), HIV prevalence was 12.27% (818/6664). Considering ineligible respondents (n=2048), 2.29% (47/2048) self-declared as cisgender women, 1.56% (32/2048) as transgender women or transvestites, 1.95% (40/2048) as transgender men, and 9.33% (191/2048) as other genders.

Among those included in this analysis (n=5065), most participants were from southeast Brazil (3532/5065, 69.73%) and accessed the survey through the Hornet app (2800/5065, 55.28%), and a total of 4618 (4618/5054, 91.37%) respondents used apps for sexual encounters. The median age of the cohort was 30 years old (interquartile range: 25-36), 63.06% (3194/5065) were white, 68.13% (3541/5065) reported middle-to-high income, and 61.36% (3106/5062) reported more than 12 years of schooling. Most respondents identified themselves as MSM (4991/5065, 98.54%) and reported having friends with the same sexual orientation (3368/5065, 66.50%). Only 20.48% (1029/5024) of respondents reported a male steady partner and 3.97% (198/4986) reported a female steady partner (Table 1).

Binge drinking and tobacco use in the last six months were reported by 71.79% (3636/5050) and 32.60% (1651/5065) of individuals, respectively (Table 2). Marijuana (or hash) was the most frequent illicit substance reported (1679/5065, 33.15%), followed by stimulants (1177/5065, 23.24%), hallucinogens (519/5065, 10.25%) and GHB (222/5065, 4.38%). A total of 2305 (2305/5065, 45.51%) participants reported no substance use in the last six months.

In the previous 6 months, only 236 (236/5065, 4.66%) respondents reported having no sexual partners. Condomless receptive anal sex prevalence was high (2121/5065, 41.88%). Approximately 10% (480/5065, 9.48%) of respondents reported having had sex with one HIV-positive partner, 2.19% (111/5064) with more than one partner, and 18.62% (943/5064) reported that they did not know how many HIV-positive partners they had sex in the prior six months. Regarding the number of times they were the insertive partner without a condom with an HIV-positive partner, 18.90% (957/5064) of participants reported one to four times and 8.87% (449/5064) reported five times or more. Reported prevalence of STIs (syphilis, gonorrhea, or rectal chlamydia) in the previous 6 months was 12.06% (604/5010; Table 2). Most of the participants (3363/5064, 66.41%) scored >10 points in *The HIV Incidence Risk for MSM Scale* (high HIV risk) and fall into the category of individuals who should undergo evaluation for PrEP use. Conversely, only 21.39% (1083/5064) of participants had high HIV risk perception and 9.16% (464/5065) reported nPEP use in the last 12 months. Among those that used PEP, 81.68% (379/464), 12.72% (59/464), 2.59% (12/464), and 3.02% (14/464) reported nPEP use once, twice, three times, and more than three times in the past 12 months (respectively).

**Table 1:** Characteristics of the individuals who completed and did not complete the questionnaire.

Parameter		Incomplete^a^, n (%)	Complete, n (%)	Total, n (%)	*P* value^b^
**Region**					.01
	North (Manaus and Belém)	72 (6.30)	185 (3.65)	257 (4.14)	
	Northeast (Salvador and Recife)	112 (9.81)	442 (8.73)	354 (8.93)	
	Central-West (Brasília and Goiânia)	102 (8.93)	500 (9.87)	602 (9.70)	
	South (Florianópolis and Porto Alegre)	90 (7.88)	406 (8.01)	496 (7.99)	
	Rio de Janeiro^c^	281 (24.61)	1225 (24.19)	1506 (24.26)	
	São Paulo^c^	485 (42.57)	2307 (45.55)	2792 (44.98)	
	Total	1142(18.40)	5065 (81.60)	6207 (100.00)	
**Apps**					<.001
	Hornet	493 (42.28)	2800 (55.28)	3293 (58.85)	
	Grindr	430 (36.88)	1867 (36.86)	2297 (36.86)	
	Other^d^	243 (20.84)	398 (7.86)	641 (10.29)	
	Total	1166 (18.71)	5065 (81.29)	6231 (100.00)	
**Age (years)**					.12
	18-24	262 (26.60)	1212 (23.93)	1474 (24.36)	
	25-35	458 (46.50)	2515 (49.65)	2973 (49.14)	
	>36	265 (26.90)	1338 (26.42)	1603 (26.50)	
	Total	985 (16.28)	5065 (83.72)	6050 (100.00)	
**Color/Race**					—
	White	—	3194 (63.06)	3194 (63.06)	
	Non-white^e^	—	1871 (36.94)	1871 (36.94)	
	Total	—	5065 (100.00)	5065 (100.00)	
**Family monthly income**^f^					—
	<3 (low income)	—	1614 (31.87)	1614 (31.87)	
	4-10 (middle income)	—	2148 (42.41)	2148 (42.41)	
	>10 (high income)	—	1303 (25.73)	1303 (25.73)	
	Total	—	5065 (100.00)	5065 (100.00)	
**Schooling (years)**					.01
	<12	391 (45.41)	1956 (38.64)	2347 (39.63)	
	>12	470 (54.59)	3106 (61.36)	3576 (60.37)	
	Total	861 (14.54)	5062 (85.46)	5923 (100.00)	
**Sexual orientation**					<.001
	MSM^g^	839 (96.11)	4991 (98.54)	5042 (84.97)	
	Other	34 (3.89)	74 (1.46)	892 (15.03)	
	Total	869 (14.64)	5065 (85.30)	5934 (100.00)	
**Friends with same sexual orientation**					.01
	Majority	433 (56.72)	3368 (66.50)	3801 (65.65)	
	A few	292 (40.28)	1697 (33.51)	1989 (34.35)	
	Total	725 (12.52)	5065 (87.48)	5790 (100.00)	


**Steady partner (male)**					.02
	Yes	205 (24.09)	1029 (20.48)	1234 (21.00)	
	No	646 (75.91)	3995 (79.52)	4641 (79.00)	
	Total	851 (14.49)	5024 (85.51)	5875 (100.00)	
**Steady partner (female)**					.08
	Yes	44 (5.28)	198 (3.97)	242 (4.16)	
	No	789 (94.72)	4788 (96.02)	5577 (95.84)	
	Total	833 (14.32)	4986 (85.68)	5819 (100.00)	
**Know someone living with HIV**					.01
	Yes	263 (31.0)	3491 (68.94)	1856 (31.72)	
	No	462 (68.94)	1573 (31.06)	3953 (68.28)	
	Total	725 (12.54)	5064 (87.48)	5789 (100.00)	
**Use of apps for sexual encounters**					.02
	Never	85 (11.74)	436 (8.63)	521 (9.02)	
	Sometimes	338 (46.69)	2564 (50.72)	2902 (50.22)	
	Daily	259 (35.77)	1798 (35.58)	2057 (35.60)	
	Only when travelling or vacations	42 (5.80)	256 (5.07)	298 (5.16)	
	Total	724 (12.53)	5054 (87.47)	5778 (100.00)	

^a^No answer for variables color and race (last questions of the questionnaire).

^b^Chi-square test.

^c^Southeast Brazil.

^d^Other social media (eg, Facebook, WhatsApp), email.

^e^Black, Asian, Native American, mixed-race, or don’t know.

^f^Brazilian monthly minimum wage in 2016 was 880 BRL (US $267, currency from July 2016).

^g^Homosexual, gay, or bisexual.

A total of 738 (738/5065, 14.57%) respondents had never performed an HIV test and one of the main reasons was the fear of having an HIV-positive result (263/738, 35.64%). Compared to those who had been tested before, these respondents were younger (24 years old, interquartile range: 21-30), had less years of schooling (<12 years: 495/736, 67.26%), had lower income (less than 4 minimum wage: 359/738, 48.64%), and almost half self-reported as white (370/738, 50.14%). Almost half of the respondents believed that the best testing venue is at home (2400/5065, 47.38%) and most would be comfortable with picking up the HIVST somewhere else (2867/5065, 56.60%; Table 3).

**Figure 2:**
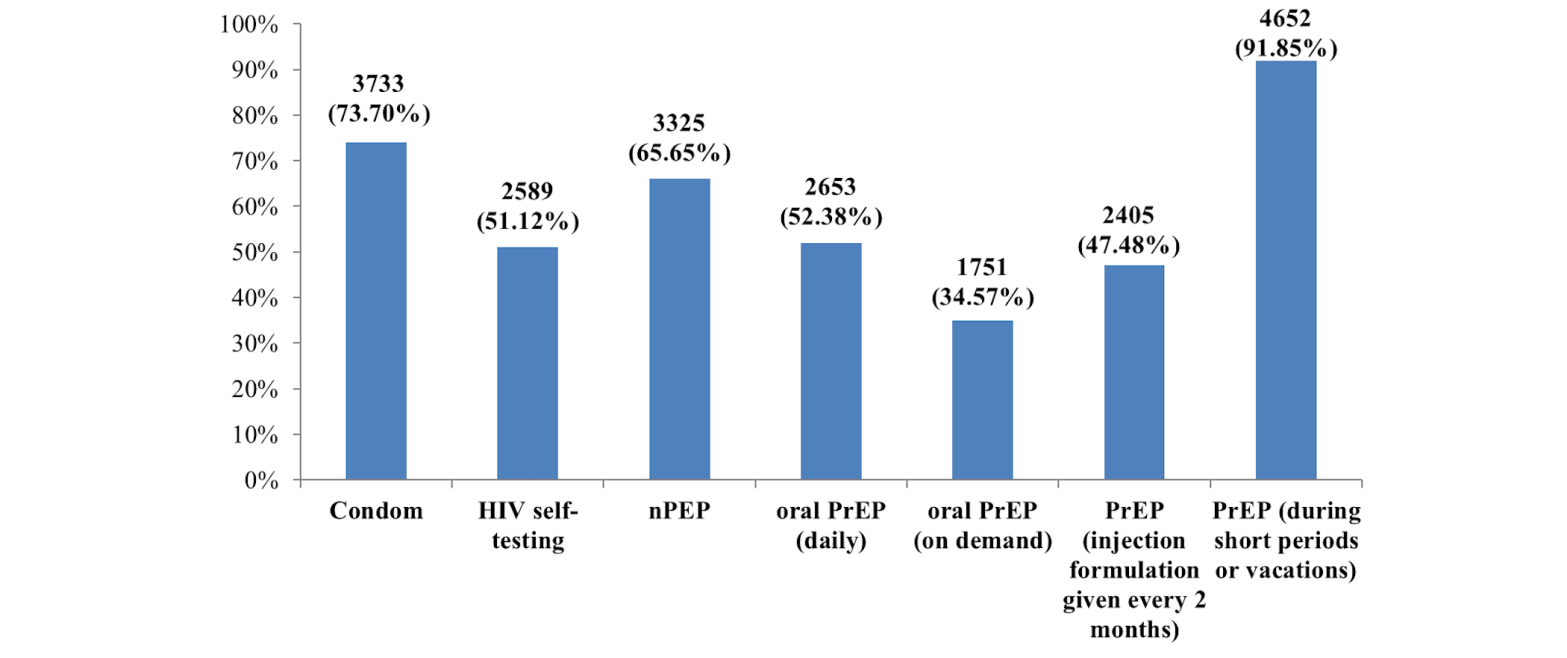
Willingness to use HIV prevention methods (n=5065). PrEP: preexposure prophylaxis; nPEP: nonoccupational postexposure prophylaxis.

PrEP, nPEP and HIVST awareness was reported by 57.89% (2932/5065), 57.39% (2907/5065), and 26.57% (1346/5065) of respondents, respectively. Willingness to use different HIV prevention methods is depicted in Figure 2. Willingness to use daily oral PrEP and injected PrEP was similar (2653/5065, 52.38% vs 2408/5065, 47.48%), while PrEP on demand was lower (1751/5065, 35.57%) and PrEP during short periods or vacations was much higher (4652/5065, 91.85%). In addition, 51.08% (2587/5065) of respondents would use PrEP if available commercially and 4.72% (239/5065) would never use PrEP.

In the final multivariate model (Multimedia Appendix 1), variables independently associated with daily oral PrEP willingness were: high number of male sexual partners (>10) versus 0-5 partners (AOR 1.26, 95% CI 1.09-1.47), condomless receptive anal intercourse (AOR 1.27, 95% CI 1.12-1.44), sex with HIV-positive partner versus no HIV-positive partner (one HIV-positive partner: AOR 1.36, 95% CI 1.11-1.67), daily use of apps for sexual encounters versus never use (AOR 1.48, 95% CI 1.17-1.87), high and unknown perceived likelihood of getting HIV in the next year (AOR 1.72, 95% CI 1.47-2.02 and AOR 1.39, 95% CI 1.13-1.70), STI diagnosis (AOR 1.25, 95% CI 1.03-1.51), stimulant use (AOR 1.24, 95% CI 1.07-1.43), PrEP awareness (AOR 1.48, 95% CI 1.30-1.70), and unwillingness to use condoms (AOR 1.16, 95% CI 1.00-1.33).

**Table 2:** Binge drinking, substance use, and risk behaviors among the study population (n=5065). MSM: men who have sex with men.

Parameter		Total, n (%)
Binge drinking^a^ (yes; n=5050)		3636 (71.79)
**Substance use**^a^		
	Tobacco	1651 (32.60)
	Stimulants^b^	1177 (23.24)
	GHB^c^	222 (4.38)
	Marijuana or hash	1679 (33.15)
	Hallucinogens^d^	519 (10.25)
**Number of male sexual partners**^a^		
	0-5	2609 (51.51)
	6-10	1001 (19.76)
	>10	1455 (28.73)
Condomless receptive anal sex^a^ (yes)		2121 (41.88)
**Number of male HIV-positive sexual partner(s)**^a^ **(n=5064)**		
	None	3530 (69.71)
	1	480 (9.48)
	>1	111 (2.19)
	Unknown	943 (18.62)
**Number of insertive condomless anal intercourse with HIV-positive partner**^a^ **(n=5064)**		
	None	3391 (66.96)
	1-4 intercourses	957 (18.90)
	5 or more intercourses	449 (8.87)
	Unknown	267 (5.27)
The HIV Incidence Risk for MSM Scale^e,f^ (>10 points; high risk)		3363 (66.41)
Money for sex^a^ (n=5045)		202 (3.99)
**Perceived likelihood of getting HIV**^g^		
	Low	3516 (69.41)
	High	1083 (21.39)
	Unknown	466 (9.20)
STI diagnosis^a,h^ (n=5010)		604 (12.06)

^a^During the previous 6 months.

^b^Cocaine, poppers, crack, or ecstasy.

^c^4-hydroxybutanoic acid.

^d^Solvents, lysergic acid diethylamide, ketamine.

^e^*The HIV Incidence Risk for MSM Scale* was calculated based on sexual behavior in the previous 6 months (number of partners, condomless receptive anal intercourse, sex with HIV-positive partner, and use of stimulants; if >10 points, PrEP is recommended).

^f^“Unknown” answers scored 0 points on *The HIV Incidence Risk for MSM Scale*.

^g^In the next 12 months.

^h^Syphilis, gonorrhea, or rectal chlamydia.

**Table 3:** Previous HIV testing and preferences (N=5065). HIVST: human immunodeficiency virus self-testing; SUS: Brazilian public health system.

Parameter		Total, n (%)
**HIV testing**		
	Never	738 (14.57)
	Once (lifetime)	762 (15.04)
	Once a year	1073 (21.18)
	More than once a year	1171 (23.12)
	Every time I am exposed	311 (6.14)
	Sporadically	1010 (19.94)
**Reasons for never testing (n=738)**		
	No risk of be infected	100 (13.55)
	Not easy to reach health care provider	50 (6.78)
	Shame	128 (17.34)
	Afraid of being positive	263 (35.64)
	Lazy	74 (10.03)
	Other	123 (16.66)
**Best place for testing**		
	At home	2400 (47.38)
	Health care provider	1987 (39.23)
	Pharmacy	468 (9.24)
	Community center	61 (1.20)
	Other	149 (2.94)
**Best way to obtain HIVST (if available at SUS)**		
	Internet (home delivery)	2094 (41.34)
	Internet (pick somewhere)	578 (11.41)
	Pick up at a pharmacy	1059 (20.91)
	Pick up at a health care provider	1126 (22.23)
	Pick up at a nongovernmental organization	104 (2.05)
	Other	104 (2.05)

## Discussion

Our findings provide important insights about the characteristics of Brazilian MSM based on two popular GSN apps for sexual encounters, and their preferences amidst the HIV prevention strategies. This information can potentially guide and support national prevention programs. Over half of the respondents would be willing to use daily oral PrEP if available through SUS, which is lower than previously reported in Brazil [21]. This finding is probably related to the fact that the previous studies also included individuals who were actively seeking PrEP, and thus possibly overestimated PrEP willingness. Nevertheless, our finding is consistent with reports from other settings [14,20-28,34] and represents a reassuring result, given that daily PrEP will soon be provided by SUS at no cost for MSM at higher risk of contracting HIV [48].

Our study showed that, in addition to daily oral PrEP, a high proportion of respondents were also interested in other PrEP formulations, such as injectable PrEP (2408/5065, 47.48%). Long-acting injectable PrEP can be very beneficial among individuals for whom adherence to a daily oral regimen is challenging [22,49,50]. Cabotegravir, an integrase inhibitor that can be administered through intramuscular injection, is one of the products currently under clinical development for PrEP formulation [51]. Individuals’ personal preferences and priorities may have a significant impact on acceptability, uptake, and retention of different PrEP modalities and the ability to choose among multiple contraceptive methods, which was shown to be associated with increased population coverage [52].

Although nPEP has been available at no cost through SUS since 2009, awareness, willingness, and use were lower than expected. This problem is highly concerning given that almost half of our sample reported unprotected anal sex. Similar results have been described from other settings with MSM-concentrated epidemics [53,54].

Few of the respondents had heard about HIVST; however, half of them would be willing to use it and this strategy, which could increase serostatus awareness and facilitate integration into HIV care [55]. The acceptability of HIVST ranged from 21-98% in studies among MSM [56], including a pilot study using blood-based HIVST in Brazil and Peru [57], and an Internet-based feasibility study using oral fluid, which was conducted in Brazil [58]. As social networking advertisements on HIVST were shown to be effective at increasing HIVST awareness and uptake [59,60], feasibility of HIVST distribution through apps for sexual encounters should also be further evaluated.

Although smartphones became the main devices used for Internet access, especially in lower income families [61], it is hard to estimate the proportion and possible selection bias of online studies. Most of the MSM included in this study were white and aged 25-36 years, and reported middle to higher income with more schooling years, which may not reflect the Brazilian MSM population. Some of these characteristics are in accordance with a systematic review, which showed that those using apps were younger, presented higher educational levels, reported higher incomes, had higher proportions of risky sexual behaviors, and were more likely to have tested for HIV in their lifetime compared to nonapp-using MSM [62]. In addition, our sample was similar to the one recruited on Grindr for a study in Los Angeles [63] and another from a social networking site for MSM in Latin America, Spain, and Portugal [38]. Indeed, studies have shown that online samples tend to be biased toward a lower median age, as younger MSM are over-represented on GSN apps [64]. Conversely, younger MSM aged 24 years or less was not the majority of this sample, perhaps because the content of the survey or the advertisement strategy was not attractive to this population.

Considering *The HIV Incidence Risk for MSM Scale*, most of the sample should be further evaluated for PrEP use, but perceived likelihood of getting HIV in next year was low. Moreover, rates of unprotected anal intercourse reported by study participants was high, which has also been found to be high among MSM who use Grindr in studies conducted in the United States [35,65] and among MSM from a website for sexual encounters in Latin America [38]. The CDC recommends that sexually active MSM should be tested annually, and clinicians should consider the potential benefits of more frequent HIV screening (eg, every three or six months) for some asymptomatic sexually active MSM, based on their individual risk factors, local HIV epidemiology, and local policies [66,67]. Almost 15% of the sample (738/5065) had never performed an HIV test and 15% (762/5065) were only tested once in their lifetime, which is almost two-fold higher than that observed in a US study using a GSN app [68], but much lower than that found in other Brazilian studies [2,69]. Knowledge of HIV status enables individuals to make decisions about behavioral strategies to reduce HIV transmission risks, such as serosorting [70-72], using condoms with partners who do not share the same HIV status, or restricting behaviors to partners who are HIV-positive and have undetectable viral loads (UVLs) or HIV-negative partners that take PrEP [73]. Disclosure of PrEP use and UVL is not uncommon among MSM using apps in the United States, and the majority of the respondents have engaged in condomless sex at least once based on this status [73]. This behavior is unknown in Brazil, as PrEP is still not available and information and knowledge about the relationship of UVL and HIV transmission is not widely spread. Hopefully this reality will change in the foreseeable future, and studies to evaluate this behavior shift are needed.

Binge drinking and tobacco and substance use were high in the study population, compared with the general population [74], which is consistent with previous reports [75,76]. In an online survey among MSM through Facebook in seven countries, including Brazil, it was observed that social networks and minority stressors can have significant effects on drug use and sex while drunk or high [77]. Binge drinking and drug use (eg, marijuana, amphetamines, poppers) in general have been associated with condomless intercourse [78-80]. A recent study in young lesbian, gay, and bisexual individuals showed that the use of marijuana is associated with sex with multiple partners [81]. Conversely, a lower prevalence of binge drinking and substance use was observed during the PrEP Brasil Study [10]. Nevertheless, public policies on HIV prevention still need to acknowledge and address the relationship between substance use and risk behaviors.

Moreover, our results provide evidence that MSM reporting higher risk behaviors were more willing to use daily PrEP, as observed in other studies [21,82-87]. Accordingly, our results show that those reporting higher or unknown perceived HIV risk and STI diagnoses in the last six months were also more willing to use daily PrEP. This is an important finding since PrEP is recommended to high-risk MSM. In addition, those not willing to use condoms were more willing to use PrEP. In a study comparing preferences for PrEP, condoms, and both PrEP and condoms, MSM reporting recent risk behaviors were more likely to prefer PrEP compared with condoms only, and less likely to prefer both methods compared with condoms only [88].

Unequivocally, this study has limitations. First, online studies are not probabilistic sampling strategies, thus precluding the generalizability of the findings. Given the cross-sectional nature of the data, causality and the direction of association may not be inferred. All collected data were self-reported by participants and may be subjected to bias, including social desirability bias. Our data were also subjected to recall bias due to 6-month or 12-month recall periods. There is also a concern about participants taking the survey multiple times. To avoid this issue, the first question of the survey was, “Are you answering this survey for the first time?” (4% of participants answered “no” and were excluded from the study). Finally, we have measured intention to use PrEP, nPEP, condoms, and HIVST as a proxy of willingness. There are different methods for accessing PrEP willingness, as reviewed by Young and McDaid [89], and as such our results should be interpreted with care.

In summary, our observed high HIV risk scores suggest that most MSM would be eligible for PrEP, and that those who present risky sexual behaviors were more willing to use it. Notwithstanding, only 58% (2932/5065) of individuals were aware of this prevention strategy. Additionally, awareness, willingness, and use of nPEP—which has been available in Brazil since 2009—were low. Efforts to increase awareness of new prevention strategies are urgently needed to create demand among those at the highest risk for HIV infection. Mobile health tools are a promising strategy to reach high risk MSM in Brazil.

## Appendix

Multimedia Appendix 1 Factors associated with daily PrEP willingness.

## Abbreviations

AIDS: acquired immune deficiency syndrome
AOR: adjusted odds ratio
CDC: Centers for Disease Control and Prevention
GHB: 4-hydroxybutanoic acid
GSN: geosocial networking
HIVST: human immunodeficiency virus self-testing
MSM: men who have sex with men
nPEP: nonoccupational postexposure prophylaxis
OR: odds ratio
PEP: postexposure prophylaxis
PrEP: preexposure prophylaxis
STI: sexually transmitted infection
SUS: Brazilian public health system
UVL: undetectable viral loads
