# Supplementary material for: Awareness of Prevention Strategies and Willingness to Use Preexposure Prophylaxis in Brazilian Men Who Have Sex With Men Using Apps for Sexual Encounters: Online Cross-Sectional Study
Source: JMIR Public Health Surveill. 2018 Jan 22;4(1):e11. doi: 10.2196/publichealth.8997 (PMC5799718; doi:10.2196/publichealth.8997)
Supplement: Multimedia Appendix 1 [file publichealth_v4i1e11_app1.pdf]

Multimedia Appendix 1. Factors associated with daily PrEP willingness. Brazil, 2016.

	Willingness to use PrEP		Bivariate models		Multivariate model
	Yes (N=2653; 52.38%)	No (N=2412; 47.62%)	OR (95% CI)	*P* value	AOR (95% CI) [a]
Age (years)				.26	
18-24	611(23.03)	601(24.92)	Ref.		Ref.
25-35	1344(50.66)	1171(48.55)	1.12(0.98, 1.29)		1.05(0.89, 1.23)
≥36	698(26.31)	640(26.53)	1.07(0.91, 1.25)		1.03(0.86, 1.24)
Color				.07	
White	1702(64.15)	1492(61.86)	1.11(0.99, 1.25)		1.10(0.97, 1.24)
Non-white [b]	951(35.85)	920(38.14)	Ref.		Ref.
Family Monthly Income [c]					
≤ 3 (low)	852(32.11)	762(31.59)	Ref.		Ref.
4-10 (middle)	1108(41.77)	1040(43.12)	0.97(0.85, 1.10)		0.91(0.79, 1.06)
>10 (high)	693(26.12)	610(25.29)	1.03(0.89, 1.19)		0.96(0.80, 1.14)

Schooling				.73	
< 12 years	1034(38.97)	922(38.25)	1.02(0.91, 1.14)		1.11(0.96, 1.29)
≥ 12 years	1618(70.03)	1488(61.75)	Ref.		Ref.
Friends with same sexual orientation				*.01*	-
Majority	1827(68.87)	1541(63.89)	*1.25(1.11, 1.41)*		-
None or a few	826(31.13)	871(36.11)	*Ref.*		-
Number of male sexual partners [c]				*<.001*	
0-5	1213(45.72)	1396(57.88)	*Ref.*		Ref.
6-10	529(19.95)	472(19.57)	*1.26(1.09, 1.46)*		1.01(0.86, 1.18)
>10	911(34.33)	544(22.55)	*1.93(1.69, 2.20)*		*1.26(1.09, 1.47)*
Condomless receptive anal sex [c]				*<.001*	
Yes	1249(47.08)	872(36.15)	*1.60(1.42, 1.79)*		*1.27(1.12, 1.44)*
No	1404(52.92)	1540(63.85)	*Ref.*		*Ref.*
Number of male HIV+ sexual partner[c]				*<.001*	

None [d]	2265(85.37)	2208(91.54)	*Ref.*		Ref.
1	305(11.50)	175(7.30)	*1.72(1.41, 2.10)*		*1.36(1.11, 1.67)*
>1	83(3.13)	28(1.16)	*2.76(1.79, 4.27)*		1.40(0.89, 2.21)
Number of Insertive condomless anal intercourses with HIV+ partner[c]				*<.001*	-
<5 [d]	2365(89.14)	2250(93.30)	*Ref.*		-
≥5	288(10.86)	161(6.70)	*1.72(1.40, 2.11)*		-
Use of apps for sexual encounters				*<.001*	
Never	180(6.79)	256(10.65)	*Ref.*		Ref.
Sometimes	1267(47.81)	1297(53.95)	*1.42(1.15, 1.75)*		1.12(0.90, 1.40)
Daily	1091(41.17)	707(29.40)	*2.20(1.77, 2.73)*		*1.48(1.17, 1.87)*
Only when travelling or vacations	112(4.23)	144(6.00)	*1.10(0.80, 1.52)*		0.91(0.66, 1.27)
Money for sex [e]				*.01*	-
Yes	123(4.66)	79(3.29)	*1.48(1.10, 1.99)*		-

No	2519(95.34)	2324(96.71)	*Ref.*		-
HIV test				*.01*	-
At least once	2303(86.81)	2024(83.91)	*1.26(1.08, 1.48)*		-
Never	350(13.19)	388(16.09)	*Ref.*		-
Perceived likelihood of getting HIV [f]				*<.001*	
Low (<50%)	1655(62.38)	1861(77.16)	*Ref.*		*Ref.*
High (≥50%)	733(27.63)	350(14.51)	*2.36(2.03, 2.73)*		*1.72(1.47, 2.02)*
Unknown	265(9.99)	201(8.33)	*1.51(1.24, 1.85)*		*1.39(1.13, 1.70)*
Know someone living with HIV				*<.001*	-
Yes	1899(71.58)	1592(66.03)	*1.33(1.17, 1.50)*		-
No	754(28.42)	819(33.97)	*Ref.*		-
STI diagnosis [e, g]				*<.001*	
Yes	384(14.68)	220(9.18)	*1.72(1.44, 2.06)*		*1.25(1.03, 1.51)*
No	2231(85.32)	2175(90.82)	*Ref.*		*Ref.*

Binge drinking [e]				*.02*	-
Yes	1942(73.42)	1694(70.44)	*1.15(1.02, 1.31)*		-
No	703(26.58)	711(29.56)	*Ref.*		-
Stimulants use [e, h]				*<.001*	
Yes	726(27.37)	451(18.70)	*1.63(1.42, 1.86)*		*1.24(1.07, 1.43)*
No	1927(72.63)	1961(81.30)	*Ref.*		*Ref.*
GHB [i]				*<.001*	-
Yes	148(5.58)	74(3.07)	*1.91(1.43, 2.56)*		-
No	2505(94.42)	2338(96.93)	*Ref.*		-
PrEP awareness				*<.001*	
Yes	1687(63.59)	1245(51.62)	*1.62(1.44, 1.82)*		*1.48(1.30, 1.70)*
No	966(36.41)	1167(48.38)	*Ref.*		*Ref.*
Willingness to use Condom				*<.001*	
Yes	1843(69.47)	1890(78.36)	*Ref.*		*Ref.*
No	810(30.53)	522(21.64)	*1.61(1.42, 1.83)*		*1.16(1.00, 1.33)*

[a] variables with $p<0.25$ in bivariate models were included in the initial multivariate model. Variables with $p<0.05$ were kept in the final multivariate model, excepted for age, color and schooling defined a priori, statistically significant associations in *Italics*; [b] Black, Asian, Native American or don't know; [c] Brazilian montly minimun wage in 2016 was 880 BRL (267 USD, currency from July 2016); [d] Including "unknown"; [e] During the previous 6 months; [f] In the next 12 months; [g] syphilis, gonorrhea or rectal chlamydia; [h] cocaine, poppers, crack or ecstasy; [i] 4-hydroxybutanoic acid.
